# Crystal structure of bis­(μ_2_-4-*tert*-butyl-2-formyl­phenolato)-1:2κ^3^ *O* ^1^,*O* ^2^:*O* ^1^;3:4κ^3^ *O* ^1^,*O* ^2^:*O* ^1^-bis­(4-*tert*-butyl-2-formyl­phenolato)-2κ^2^ *O* ^1^,*O* ^2^;4κ^2^ *O* ^1^,*O* ^2^-di-μ_3_-methoxido-1:2:3κ^3^ *O*;1:3:4κ^3^ *O*-di-μ_2_-methoxido-1:4κ^2^ *O*;2:3κ^2^ *O*-tetra­copper(II)

**DOI:** 10.1107/S205698901500376X

**Published:** 2015-02-28

**Authors:** Bernhard Eberhard Christian Bugenhagen, Marc Heinrich Prosenc

**Affiliations:** a Institute for Inorganic and Applied Chemistry, University of Hamburg, Martin-Luther-King-Platz 6, D-20146 Hamburg, Germany; b TU Kaiserslautern, Physikalische Chemie, Erwin-Schrödinger-Strasse 52, D-67663 Kaiserslautern, Germany

**Keywords:** crystal structure, copper(II), tetra­nuclear complex, hydrogen bonding

## Abstract

The structure of the title compound, [Cu_4_(CH_3_O)_4_(C_11_H_13_O_2_)_4_], consists of dimeric dinuclear copper(II) complexes oriented around a centre of inversion. Within each dinuclear fragment, the two Cu^II^ atoms are in a distorted square-planar coordination sphere. Two neighbouring fragments are linked by four apical Cu—O contacts, yielding an overall square-pyramidal coordination environment for each of the four Cu^II^ atoms. The mol­ecules are arranged in layers parallel to (101). Non-classical C—H⋯O hydrogen-bonding inter­actions are observed between the layers.

## Abstract 2

The dinuclear title compound crystallizes as a dimer forming a tetra­nuclear copper(II) complex, [Cu_4_(CH_3_O)_4_(C_11_H_13_O_2_)_4_], in the solid state. In this complex, all Cu^II^ atoms have a square-pyramidal coordination sphere, with long axial and short basal Cu—O distances.

## Chemical context   

The title compound was obtained as a by-product in the synthesis of an unsymmetrically substituted copper(II) salophene complex (Kleij *et al.*, 2005 ▸). The latter is of inter­est with respect to magnetic properties and cooperative effects between the metal(II) atoms (Kahn *et al.*, 1982 ▸). In this compound, three types of bridging oxygen ligands are found. The magnetic exchange coupling between the paramagnetic Cu^II^ atoms is considered as strong in this type of bridges since the Cu—O—Cu angles are found to be close to 90°. The distances and coordination modes between Cu^II^ atoms vary and thus, the compound is a suitable study case for investigating different spin-coupling paths. This knowledge is deemed important for the design of tailor-made magnetic compounds.
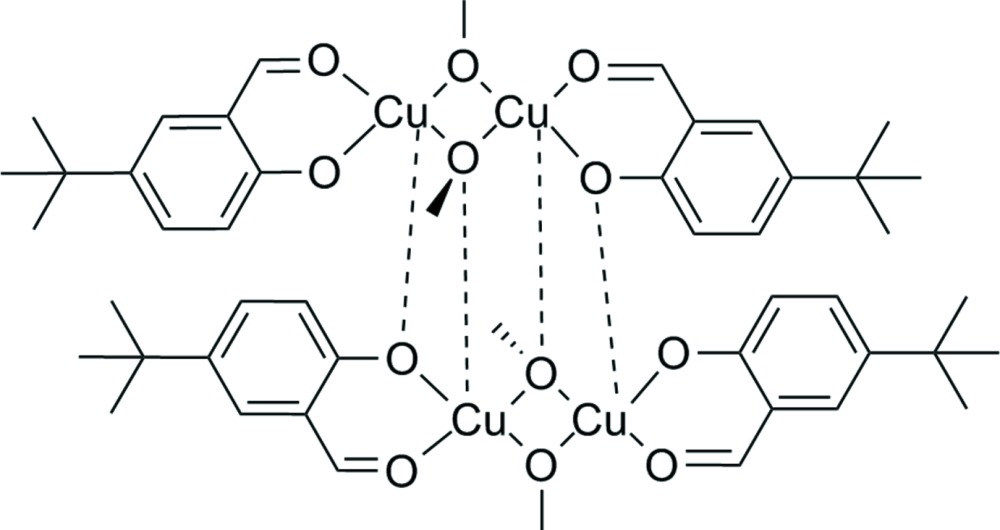


## Structural commentary   

**Figure 1:**
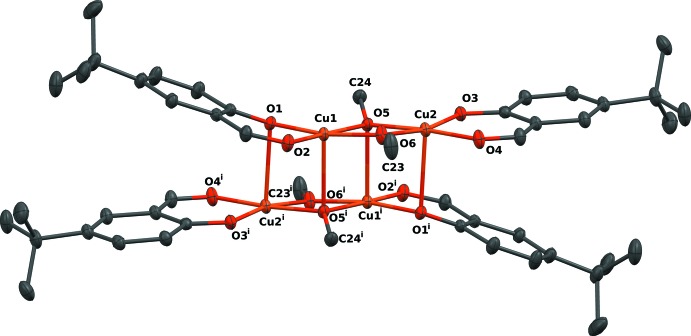
The tetra­nuclear mol­ecule in the title compound. Displacement ellipsoids are shown at the 50% probability level. [Symmetry code: (i) −*x* + 2, −*y* + 1, −*z*.]

The tetra­nuclear copper(II) title compound consists of two dinuclear complex fragments oriented around a centre of inversion. Within each fragment the two Cu^II^ atoms are in a distorted square-planar coordination sphere, thereby bridged by two κ^2^ *O* methoxido ligands. The terminal bidentate 4-*tert*-butyl-2-formyl­phenolate ligand is coordinating each Cu^II^ atom in a manner generating a pseudo-mirror plane perpendicular to the four-membered bis-methoxido dicopper ring in the centre of the fragment. A longer Cu—O bond completes the overall square-pyramidal coordination for each Cu^II^ atom and links the two dinuclear fragments together. The distance between the two copper(II) ions Cu1 and Cu2 within the binuclear fragment is 2.9938 (2) Å (Fig. 1 ▸) which is in the same range as in related complexes (Kahn *et al.*, 1982 ▸).

Short distances Cu1—O1 of 1.9166 (8) Å, Cu1—O2 of 1.9557 (9) Å, Cu1—O5 of 1.9522 (8) Å and Cu1—O6 of 1.9154 (9) Å are found for the Cu1 atom to the basal O atoms within the binuclear fragment. A substanti­ally longer distance of 2.3703 (9) Å is observed for the apical Cu1—O5^i^ [symmetry code: (i) −*x* + 2, −*y* + 1, −*z*] bond to the methoxido ligand of the neighbouring fragment. For the Cu2 atom, the situation is comparable, with slightly shorter Cu—O distances in comparison with Cu1: Cu2—O3 1.8939 (8) Å, Cu2—O4 1.9473 (9) Å, Cu2—O5 1.9455 (8) Å and Cu2—O6 1.9081 (8) Å. The longer distance Cu2—O1^i^ of 2.4994 (9) Å to the phenoxido ligand atom of the neighbouring fragment causes less sterical congestions at the Cu2 atom and thus, appears to be the cause for the shorter basal Cu—O distances.

The binding modes (μ_2_ *versus* μ_3_) of the two methoxido ligands in each fragment can be distinguished by the angles C24—O5—O6 [152.62 (8)°, μ_3_] *versus* C23—O6—O5 [173.52 (11)°, μ_2_] (Fig. 1 ▸). Meth­oxy ligand atom O5 is more closely bound to the Cu1^i^ atom, in addition with two short distances to Cu1 and Cu2 (see above), resulting in a more pyramidal-like geometry. This differs to the more trigonal-planar geometry of O6 (see Table 1 ▸ and Fig. 1 ▸) which is not bound to a third Cu atom but has two short distances to Cu1 and Cu2. In the salicyl­aldehyde ligands, the presence of a second metal ion coordinated by the phenoxide O atom has an effect on the phen­yl—O bond length, which is slightly elongated compared to the one in the non-bridging salicyl­aldehyde ligand [1.3075 (13) Å *versus* 1.2963 (13) Å].

Within the dinuclear fragment, the aromatic rings are tilted by an angle of 24.69 (6)° due to repulsion of the *tert*-butyl groups.

## Supra­molecular features   

**Figure 2:**
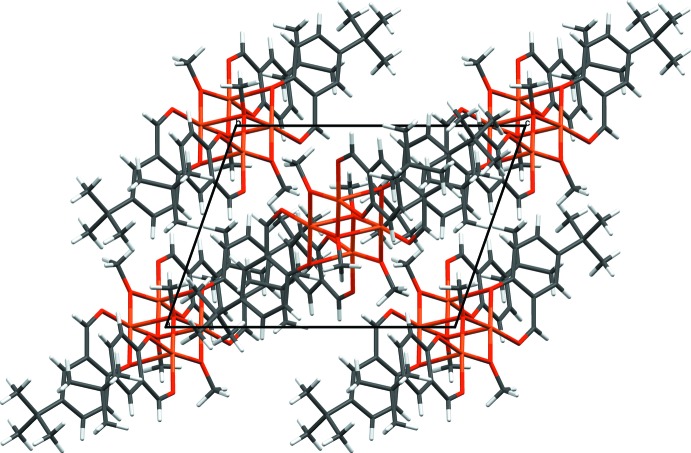
A packing diagram of the title compound.

In the crystal, the tetra­nuclear mol­ecules arrange in layers parallel to (101) (Fig. 2 ▸). Weak non-classical C—H⋯O inter­actions between the layers (Table 2 ▸) help to stabilize the crystal packing.

## Synthesis and crystallization   

After treatment of 102 mg (0.35 mmol) 4-Br-salicyl-2-(2-amino)­phenyl­imine with 113 mg of copper(II)acetate monohydrate (0.445 mmol), 1 ml tri­ethyl­amine in 10 ml THF, and 65.5 mg (0.368 mmol) 4-*tert*-butyl­salicyl­aldehyde in 10 ml THF, the mixture was stirred for 22 h at room temperature. Addition of hexane yielded the title compound as a dark crystalline material from the reaction mixture (11.7 mg, 0.011 mmol, 8%).

## Refinement   

Crystal data, data collection and structure refinement details are summarized in Table 3 ▸. The positions of all H atoms were calculated according to the geometry of the parent C atom and refined using a riding model with C—H distances of 0.95 Å and *U*_iso_(H) = 1.5*U*_eq_(C) for *sp* ^2^ C atoms and of 0.98 Å and *U*_iso_(H) = 1.5*U*_eq_(C) for *sp* ^3^ C atoms.

## Supplementary Material

Crystal structure: contains datablock(s) general, I. DOI: 10.1107/S205698901500376X/wm5122sup1.cif

Structure factors: contains datablock(s) I. DOI: 10.1107/S205698901500376X/wm5122Isup2.hkl

CCDC reference: 1050914

Additional supporting information:  crystallographic information; 3D view; checkCIF report

## Figures and Tables

**Table 1:** Selected bond angles ()

O3Cu2O5	92.75(4)	O5Cu1O2	171.62(4)
O3Cu2O4	94.19(4)	O1Cu1O5^i^	88.65(3)
O3Cu2O6	167.56(4)	O1Cu1O5	94.74(3)
O6Cu2O4	95.13(4)	O1Cu1O2	93.39(4)
O5Cu1O5^i^	84.34(3)	O2Cu1O5^i^	97.91(3)

Symmetry code: (i) 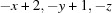.

**Table 2:** Hydrogen-bond geometry (, )

*D*H*A*	*D*H	H*A*	*D* *A*	*D*H*A*
C14H14O4^ii^	0.95	2.57	3.3225(16)	136
C23H23*B*O2	0.98	2.43	3.0607(18)	122

Symmetry code: (ii) 
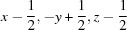
.

**Table 3:** Experimental details

Crystal data	
Chemical formula	[Cu_4_(CH_3_O)_4_(C_11_H_13_O_2_)_4_]
*M*_r_	1087.15
Crystal system, space group	Monoclinic, *P*2_1_/*n*
Temperature (K)	100
*a*, *b*, *c* ()	9.6863(1), 20.8460(2), 13.1387(1)
()	109.29
*V* (^3^)	2504.05(4)
*Z*	2
Radiation type	Mo *K*
(mm^1^)	1.73
Crystal size (mm)	0.10 0.10 0.10

Data collection	
Diffractometer	Bruker APEXII CCD
Absorption correction	Multi-scan (*SADABS*; Bruker, 2009 ▸)
*T*_min_, *T*_max_	0.919, 1
No. of measured, independent and observed [*I* > 2(*I*)] reflections	105316, 9505, 7960
*R*_int_	0.036
(sin /)_max_ (^1^)	0.769

Refinement	
*R*[*F* ^2^ > 2(*F* ^2^)], *wR*(*F* ^2^), *S*	0.026, 0.071, 1.00
No. of reflections	9505
No. of parameters	297
H-atom treatment	H-atom parameters constrained
_max_, _min_ (e ^3^)	1.02, 0.32

Computer programs: *APEX2* and *SAINT* (Bruker, 2009 ▸), *SHELXS98* and *SHELXL98* (Sheldrick, 2008 ▸) and *OLEX2* (Dolomanov *et al.*, 2009 ▸).

## supplementary crystallographic information

### Crystal data

**Table:**

[Cu_4_(CH_3_O)_4_(C_11_H_13_O_2_)_4_]	*F*(000) = 1128
*M**_r_* = 1087.15	*D*_x_ = 1.442 Mg m^−^^3^
Monoclinic, *P*2_1_/*n*	Mo *K*α radiation, λ = 0.71073 Å
*a* = 9.6863 (1) Å	Cell parameters from 32205 reflections
*b* = 20.8460 (2) Å	θ = 2.3–37.5°
*c* = 13.1387 (1) Å	µ = 1.73 mm^−^^1^
β = 109.29°	*T* = 100 K
*V* = 2504.05 (4) Å^3^	Rhomb, green
*Z* = 2	0.10 × 0.10 × 0.10 mm

### Data collection

**Table:**

Bruker APEXII CCD diffractometer	7960 reflections with *I* > 2σ(*I*)
Radiation source: micro-focus	*R*_int_ = 0.036
φ and ω scans	θ_max_ = 33.1°, θ_min_ = 1.9°
Absorption correction: multi-scan (*SADABS*; Bruker, 2009)	*h* = −14→14
*T*_min_ = 0.919, *T*_max_ = 1	*k* = −32→32
105316 measured reflections	*l* = −20→19
9505 independent reflections	

### Refinement

**Table:**

Refinement on *F*^2^	Primary atom site location: structure-invariant direct methods
Least-squares matrix: full	Hydrogen site location: inferred from neighbouring sites
*R*[*F*^2^ > 2σ(*F*^2^)] = 0.026	H-atom parameters constrained
*wR*(*F*^2^) = 0.071	*w* = 1/[σ^2^(*F*_o_^2^) + (0.0364*P*)^2^ + 1.1362*P*] where *P* = (*F*_o_^2^ + 2*F*_c_^2^)/3
*S* = 1.00	(Δ/σ)_max_ = 0.002
9505 reflections	Δρ_max_ = 1.02 e Å^−^^3^
297 parameters	Δρ_min_ = −0.32 e Å^−^^3^
0 restraints	

### Special details

**Table:**

Geometry. All e.s.d.'s (except the e.s.d. in the dihedral angle between two l.s. planes) are estimated using the full covariance matrix. The cell e.s.d.'s are taken into account individually in the estimation of e.s.d.'s in distances, angles and torsion angles; correlations between e.s.d.'s in cell parameters are only used when they are defined by crystal symmetry. An approximate (isotropic) treatment of cell e.s.d.'s is used for estimating e.s.d.'s involving l.s. planes.

### Fractional atomic coordinates and isotropic or equivalent isotropic displacement parameters (Å^2^)

**Table:**

	*x*	*y*	*z*	*U*_iso_*/*U*_eq_	
Cu2	1.13038 (2)	0.38778 (2)	0.05553 (2)	0.01384 (4)	
Cu1	1.00190 (2)	0.50442 (2)	0.12264 (2)	0.01311 (4)	
O5	0.94839 (9)	0.43609 (4)	0.01506 (7)	0.01477 (15)	
O1	0.81072 (9)	0.54209 (4)	0.07888 (7)	0.01482 (15)	
O2	1.08134 (10)	0.56551 (4)	0.24050 (7)	0.01891 (17)	
O3	1.04828 (9)	0.32248 (4)	−0.04680 (7)	0.01587 (15)	
O4	1.32461 (10)	0.34946 (4)	0.10451 (7)	0.01933 (17)	
C17	1.26704 (13)	0.25959 (5)	−0.01668 (9)	0.01470 (19)	
C12	1.11526 (12)	0.27283 (5)	−0.06732 (9)	0.01403 (19)	
C1	0.76032 (13)	0.57743 (5)	0.14096 (9)	0.01445 (19)	
C6	0.84991 (13)	0.60899 (5)	0.23581 (10)	0.01488 (19)	
C15	1.24880 (14)	0.15844 (5)	−0.11608 (10)	0.0174 (2)	
C16	1.33003 (14)	0.20284 (6)	−0.04410 (10)	0.0175 (2)	
H16	1.4320	0.1957	−0.0112	0.021*	
C18	1.35947 (14)	0.30001 (6)	0.06423 (10)	0.0186 (2)	
H18	1.4598	0.2882	0.0912	0.022*	
C7	1.00511 (13)	0.60300 (6)	0.27384 (10)	0.0180 (2)	
H7	1.0566	0.6307	0.3312	0.022*	
C14	1.09802 (14)	0.17270 (6)	−0.16606 (10)	0.0176 (2)	
H14	1.0390	0.1430	−0.2168	0.021*	
C13	1.03372 (13)	0.22738 (6)	−0.14445 (10)	0.0171 (2)	
H13	0.9329	0.2350	−0.1817	0.021*	
C24	0.80626 (13)	0.40993 (6)	−0.03192 (10)	0.0180 (2)	
H24A	0.8022	0.3853	−0.0964	0.027*	
H24B	0.7344	0.4448	−0.0519	0.027*	
H24C	0.7840	0.3816	0.0202	0.027*	
C5	0.78601 (13)	0.64841 (6)	0.29670 (10)	0.0174 (2)	
H5	0.8485	0.6703	0.3581	0.021*	
C4	0.63767 (13)	0.65593 (6)	0.27013 (11)	0.0191 (2)	
C2	0.60768 (14)	0.58699 (6)	0.11255 (11)	0.0206 (2)	
H2	0.5438	0.5674	0.0492	0.025*	
C19	1.30872 (15)	0.09499 (6)	−0.14322 (11)	0.0223 (2)	
C8	0.56409 (15)	0.69417 (7)	0.33745 (12)	0.0246 (3)	
C3	0.55021 (14)	0.62421 (7)	0.17531 (12)	0.0238 (3)	
H3	0.4470	0.6289	0.1541	0.029*	
C9	0.67686 (17)	0.72717 (8)	0.43383 (13)	0.0312 (3)	
H9A	0.7387	0.6946	0.4812	0.047*	
H9B	0.6260	0.7520	0.4740	0.047*	
H9C	0.7379	0.7560	0.4078	0.047*	
C21	1.23665 (18)	0.03915 (6)	−0.10276 (14)	0.0311 (3)	
H21A	1.2604	0.0424	−0.0244	0.047*	
H21B	1.2733	−0.0017	−0.1205	0.047*	
H21C	1.1303	0.0411	−0.1376	0.047*	
C22	1.27128 (19)	0.08979 (7)	−0.26595 (12)	0.0314 (3)	
H22A	1.1649	0.0917	−0.3007	0.047*	
H22B	1.3082	0.0490	−0.2837	0.047*	
H22C	1.3169	0.1254	−0.2918	0.047*	
C11	0.4717 (2)	0.64811 (9)	0.38021 (15)	0.0387 (4)	
H11A	0.3980	0.6273	0.3194	0.058*	
H11B	0.4230	0.6723	0.4226	0.058*	
H11C	0.5354	0.6154	0.4259	0.058*	
C20	1.47517 (17)	0.08965 (7)	−0.09101 (14)	0.0320 (3)	
H20A	1.5224	0.1251	−0.1159	0.048*	
H20B	1.5085	0.0487	−0.1114	0.048*	
H20C	1.5011	0.0917	−0.0124	0.048*	
C10	0.46362 (19)	0.74557 (8)	0.26739 (15)	0.0382 (4)	
H10A	0.5208	0.7739	0.2371	0.057*	
H10B	0.4195	0.7708	0.3116	0.057*	
H10C	0.3863	0.7249	0.2087	0.057*	
O6	1.17413 (10)	0.45210 (4)	0.16493 (7)	0.01948 (17)	
C23	1.31150 (16)	0.46434 (7)	0.24317 (13)	0.0322 (3)	
H23A	1.3371	0.4290	0.2953	0.048*	
H23B	1.3070	0.5045	0.2807	0.048*	
H23C	1.3859	0.4680	0.2079	0.048*	

### Atomic displacement parameters (Å^2^)

**Table:**

	*U*^11^	*U*^22^	*U*^33^	*U*^12^	*U*^13^	*U*^23^
Cu2	0.01494 (7)	0.01153 (6)	0.01431 (7)	0.00242 (4)	0.00386 (5)	−0.00174 (4)
Cu1	0.01398 (7)	0.01176 (6)	0.01379 (7)	0.00176 (4)	0.00484 (5)	−0.00189 (4)
O5	0.0127 (3)	0.0129 (3)	0.0187 (4)	−0.0002 (3)	0.0052 (3)	−0.0033 (3)
O1	0.0153 (4)	0.0148 (3)	0.0154 (4)	0.0016 (3)	0.0064 (3)	−0.0025 (3)
O2	0.0165 (4)	0.0197 (4)	0.0196 (4)	0.0029 (3)	0.0047 (3)	−0.0058 (3)
O3	0.0157 (4)	0.0129 (3)	0.0185 (4)	0.0012 (3)	0.0049 (3)	−0.0023 (3)
O4	0.0188 (4)	0.0167 (4)	0.0186 (4)	0.0045 (3)	0.0009 (3)	−0.0052 (3)
C17	0.0160 (5)	0.0131 (4)	0.0136 (5)	0.0017 (4)	0.0029 (4)	−0.0010 (4)
C12	0.0161 (5)	0.0127 (4)	0.0135 (5)	0.0006 (4)	0.0052 (4)	0.0008 (3)
C1	0.0166 (5)	0.0119 (4)	0.0164 (5)	0.0001 (4)	0.0075 (4)	−0.0010 (4)
C6	0.0159 (5)	0.0132 (4)	0.0167 (5)	0.0000 (4)	0.0069 (4)	−0.0018 (4)
C15	0.0208 (5)	0.0138 (5)	0.0169 (5)	0.0030 (4)	0.0053 (4)	−0.0016 (4)
C16	0.0187 (5)	0.0151 (5)	0.0169 (5)	0.0040 (4)	0.0034 (4)	−0.0012 (4)
C18	0.0174 (5)	0.0176 (5)	0.0179 (5)	0.0044 (4)	0.0018 (4)	−0.0025 (4)
C7	0.0172 (5)	0.0179 (5)	0.0182 (5)	0.0004 (4)	0.0048 (4)	−0.0050 (4)
C14	0.0210 (5)	0.0139 (5)	0.0170 (5)	−0.0004 (4)	0.0048 (4)	−0.0025 (4)
C13	0.0164 (5)	0.0154 (5)	0.0177 (5)	0.0003 (4)	0.0031 (4)	−0.0017 (4)
C24	0.0146 (5)	0.0169 (5)	0.0227 (6)	−0.0030 (4)	0.0066 (4)	−0.0054 (4)
C5	0.0190 (5)	0.0159 (5)	0.0181 (5)	−0.0004 (4)	0.0074 (4)	−0.0050 (4)
C4	0.0178 (5)	0.0185 (5)	0.0234 (6)	−0.0009 (4)	0.0101 (4)	−0.0065 (4)
C2	0.0153 (5)	0.0228 (6)	0.0238 (6)	−0.0008 (4)	0.0066 (4)	−0.0086 (4)
C19	0.0255 (6)	0.0154 (5)	0.0245 (6)	0.0037 (4)	0.0060 (5)	−0.0052 (4)
C8	0.0208 (6)	0.0257 (6)	0.0307 (7)	−0.0018 (5)	0.0131 (5)	−0.0121 (5)
C3	0.0149 (5)	0.0273 (6)	0.0303 (7)	−0.0008 (5)	0.0088 (5)	−0.0110 (5)
C9	0.0269 (7)	0.0343 (7)	0.0351 (8)	−0.0009 (6)	0.0138 (6)	−0.0185 (6)
C21	0.0357 (8)	0.0146 (5)	0.0403 (8)	0.0035 (5)	0.0090 (6)	0.0005 (5)
C22	0.0397 (8)	0.0278 (7)	0.0271 (7)	0.0032 (6)	0.0117 (6)	−0.0116 (5)
C11	0.0411 (9)	0.0407 (9)	0.0477 (10)	−0.0119 (7)	0.0328 (8)	−0.0192 (7)
C20	0.0277 (7)	0.0237 (6)	0.0412 (9)	0.0087 (5)	0.0069 (6)	−0.0094 (6)
C10	0.0335 (8)	0.0349 (8)	0.0453 (10)	0.0127 (6)	0.0119 (7)	−0.0121 (7)
O6	0.0181 (4)	0.0183 (4)	0.0179 (4)	0.0058 (3)	0.0003 (3)	−0.0057 (3)
C23	0.0245 (7)	0.0295 (7)	0.0308 (7)	0.0102 (5)	−0.0069 (5)	−0.0140 (6)

### Geometric parameters (Å, º)

**Table:**

Cu2—Cu1	2.9938 (2)	C24—H24C	0.9800
Cu2—O5	1.9455 (8)	C5—H5	0.9500
Cu2—O3	1.8939 (8)	C5—C4	1.3710 (17)
Cu2—O4	1.9473 (9)	C4—C8	1.5303 (17)
Cu2—O6	1.9081 (8)	C4—C3	1.4177 (18)
Cu2—O1^i^	2.4994 (9)	C2—H2	0.9500
Cu1—O5^i^	2.3703 (9)	C2—C3	1.3766 (17)
Cu1—O5	1.9522 (8)	C19—C21	1.540 (2)
Cu1—O1	1.9166 (8)	C19—C22	1.535 (2)
Cu1—O2	1.9557 (9)	C19—C20	1.534 (2)
Cu1—O6	1.9154 (9)	C8—C9	1.535 (2)
O5—C24	1.4186 (14)	C8—C11	1.540 (2)
O5—O6	2.4342 (12)	C8—C10	1.533 (2)
O1—C1	1.3075 (13)	C3—H3	0.9500
O2—C7	1.2497 (14)	C9—H9A	0.9800
O3—C12	1.2963 (13)	C9—H9B	0.9800
O4—C18	1.2550 (14)	C9—H9C	0.9800
C17—C12	1.4262 (16)	C21—H21A	0.9800
C17—C16	1.4307 (16)	C21—H21B	0.9800
C17—C18	1.4187 (16)	C21—H21C	0.9800
C12—C13	1.4211 (16)	C22—H22A	0.9800
C1—C6	1.4228 (16)	C22—H22B	0.9800
C1—C2	1.4143 (17)	C22—H22C	0.9800
C6—C7	1.4244 (17)	C11—H11A	0.9800
C6—C5	1.4232 (16)	C11—H11B	0.9800
C15—C16	1.3705 (16)	C11—H11C	0.9800
C15—C14	1.4209 (17)	C20—H20A	0.9800
C15—C19	1.5331 (17)	C20—H20B	0.9800
C16—H16	0.9500	C20—H20C	0.9800
C18—H18	0.9500	C10—H10A	0.9800
C7—H7	0.9500	C10—H10B	0.9800
C14—H14	0.9500	C10—H10C	0.9800
C14—C13	1.3728 (16)	O6—C23	1.4102 (16)
C13—H13	0.9500	C23—H23A	0.9800
C24—H24A	0.9800	C23—H23B	0.9800
C24—H24B	0.9800	C23—H23C	0.9800
			
O5—Cu2—Cu1	39.90 (2)	H24A—C24—H24C	109.5
O5—Cu2—O4	172.71 (4)	H24B—C24—H24C	109.5
O3—Cu2—Cu1	132.45 (3)	C6—C5—H5	118.8
O3—Cu2—O5	92.75 (4)	C4—C5—C6	122.46 (11)
O3—Cu2—O4	94.19 (4)	C4—C5—H5	118.8
O3—Cu2—O6	167.56 (4)	C5—C4—C8	124.16 (11)
O4—Cu2—Cu1	133.32 (3)	C5—C4—C3	116.24 (11)
O6—Cu2—Cu1	38.55 (3)	C3—C4—C8	119.58 (11)
O6—Cu2—O5	78.34 (4)	C1—C2—H2	119.5
O6—Cu2—O4	95.13 (4)	C3—C2—C1	121.04 (11)
O5^i^—Cu1—Cu2	89.49 (2)	C3—C2—H2	119.5
O5—Cu1—Cu2	39.74 (2)	C15—C19—C21	108.80 (11)
O5—Cu1—O5^i^	84.34 (3)	C15—C19—C22	109.21 (11)
O5—Cu1—O2	171.62 (4)	C15—C19—C20	112.42 (11)
O1—Cu1—Cu2	134.34 (3)	C22—C19—C21	109.42 (12)
O1—Cu1—O5^i^	88.65 (3)	C20—C19—C21	108.68 (12)
O1—Cu1—O5	94.74 (3)	C20—C19—C22	108.26 (13)
O1—Cu1—O2	93.39 (4)	C4—C8—C9	111.71 (11)
O2—Cu1—Cu2	131.99 (3)	C4—C8—C11	108.89 (11)
O2—Cu1—O5^i^	97.91 (3)	C4—C8—C10	109.96 (12)
O6—Cu1—Cu2	38.38 (3)	C9—C8—C11	108.63 (13)
O6—Cu1—O5	78.00 (4)	C10—C8—C9	108.64 (12)
O6—Cu1—O5^i^	98.18 (4)	C10—C8—C11	108.95 (14)
O6—Cu1—O1	169.41 (4)	C4—C3—H3	118.4
O6—Cu1—O2	93.66 (4)	C2—C3—C4	123.15 (12)
Cu2—O5—Cu1^i^	94.89 (3)	C2—C3—H3	118.4
Cu2—O5—Cu1	100.36 (4)	C8—C9—H9A	109.5
Cu2—O5—O6	50.15 (3)	C8—C9—H9B	109.5
Cu1—O5—Cu1^i^	95.66 (3)	C8—C9—H9C	109.5
Cu1—O5—O6	50.33 (3)	H9A—C9—H9B	109.5
Cu1^i^—O5—O6	101.01 (4)	H9A—C9—H9C	109.5
C24—O5—Cu2	125.55 (7)	H9B—C9—H9C	109.5
C24—O5—Cu1^i^	106.36 (7)	C19—C21—H21A	109.5
C24—O5—Cu1	125.65 (7)	C19—C21—H21B	109.5
C24—O5—O6	152.62 (8)	C19—C21—H21C	109.5
Cu1—O1—Cu2^i^	91.61 (3)	H21A—C21—H21B	109.5
C1—O1—Cu2^i^	109.30 (7)	H21A—C21—H21C	109.5
C1—O1—Cu1	124.52 (7)	H21B—C21—H21C	109.5
C7—O2—Cu1	124.15 (8)	C19—C22—H22A	109.5
C12—O3—Cu2	126.86 (8)	C19—C22—H22B	109.5
C18—O4—Cu2	124.25 (8)	C19—C22—H22C	109.5
C12—C17—C16	120.14 (10)	H22A—C22—H22B	109.5
C18—C17—C12	122.20 (10)	H22A—C22—H22C	109.5
C18—C17—C16	117.62 (10)	H22B—C22—H22C	109.5
O3—C12—C17	124.55 (10)	C8—C11—H11A	109.5
O3—C12—C13	118.83 (10)	C8—C11—H11B	109.5
C13—C12—C17	116.62 (10)	C8—C11—H11C	109.5
O1—C1—C6	124.16 (10)	H11A—C11—H11B	109.5
O1—C1—C2	119.21 (10)	H11A—C11—H11C	109.5
C2—C1—C6	116.61 (10)	H11B—C11—H11C	109.5
C1—C6—C7	122.30 (10)	C19—C20—H20A	109.5
C1—C6—C5	120.44 (11)	C19—C20—H20B	109.5
C5—C6—C7	117.25 (11)	C19—C20—H20C	109.5
C16—C15—C14	116.48 (10)	H20A—C20—H20B	109.5
C16—C15—C19	124.61 (11)	H20A—C20—H20C	109.5
C14—C15—C19	118.90 (11)	H20B—C20—H20C	109.5
C17—C16—H16	118.8	C8—C10—H10A	109.5
C15—C16—C17	122.46 (11)	C8—C10—H10B	109.5
C15—C16—H16	118.8	C8—C10—H10C	109.5
O4—C18—C17	127.79 (11)	H10A—C10—H10B	109.5
O4—C18—H18	116.1	H10A—C10—H10C	109.5
C17—C18—H18	116.1	H10B—C10—H10C	109.5
O2—C7—C6	127.53 (11)	Cu2—O6—Cu1	103.07 (4)
O2—C7—H7	116.2	Cu2—O6—O5	51.51 (3)
C6—C7—H7	116.2	Cu1—O6—O5	51.67 (3)
C15—C14—H14	118.5	C23—O6—Cu2	126.69 (8)
C13—C14—C15	123.05 (11)	C23—O6—Cu1	129.06 (8)
C13—C14—H14	118.5	C23—O6—O5	173.52 (11)
C12—C13—H13	119.4	O6—C23—H23A	109.5
C14—C13—C12	121.19 (11)	O6—C23—H23B	109.5
C14—C13—H13	119.4	O6—C23—H23C	109.5
O5—C24—H24A	109.5	H23A—C23—H23B	109.5
O5—C24—H24B	109.5	H23A—C23—H23C	109.5
O5—C24—H24C	109.5	H23B—C23—H23C	109.5
H24A—C24—H24B	109.5		
			
Cu2^i^—O1—C1—C6	−86.12 (12)	C16—C17—C18—O4	−175.35 (13)
Cu2^i^—O1—C1—C2	92.26 (11)	C16—C15—C14—C13	−0.50 (19)
Cu2—O3—C12—C17	−1.66 (17)	C16—C15—C19—C21	113.59 (15)
Cu2—O3—C12—C13	177.57 (8)	C16—C15—C19—C22	−127.03 (14)
Cu2—O4—C18—C17	−5.2 (2)	C16—C15—C19—C20	−6.85 (19)
Cu1—Cu2—O3—C12	−178.29 (8)	C18—C17—C12—O3	1.37 (19)
Cu1—O1—C1—C6	19.97 (15)	C18—C17—C12—C13	−177.88 (11)
Cu1—O1—C1—C2	−161.65 (9)	C18—C17—C16—C15	175.79 (12)
Cu1—O2—C7—C6	0.60 (19)	C7—C6—C5—C4	−177.61 (12)
O5—Cu2—O3—C12	177.23 (9)	C14—C15—C16—C17	2.34 (19)
O1—C1—C6—C7	−2.07 (18)	C14—C15—C19—C21	−64.86 (15)
O1—C1—C6—C5	177.80 (11)	C14—C15—C19—C22	54.53 (16)
O1—C1—C2—C3	−179.56 (12)	C14—C15—C19—C20	174.71 (13)
O3—C12—C13—C14	−177.39 (11)	C5—C6—C7—O2	170.95 (12)
O4—Cu2—O3—C12	−0.55 (10)	C5—C4—C8—C9	4.2 (2)
C17—C12—C13—C14	1.90 (17)	C5—C4—C8—C11	−115.78 (15)
C12—C17—C16—C15	−2.07 (19)	C5—C4—C8—C10	124.91 (15)
C12—C17—C18—O4	2.5 (2)	C5—C4—C3—C2	0.8 (2)
C1—C6—C7—O2	−9.2 (2)	C2—C1—C6—C7	179.51 (12)
C1—C6—C5—C4	2.52 (18)	C2—C1—C6—C5	−0.62 (17)
C1—C2—C3—C4	1.0 (2)	C19—C15—C16—C17	−176.13 (12)
C6—C1—C2—C3	−1.06 (19)	C19—C15—C14—C13	178.07 (12)
C6—C5—C4—C8	175.75 (12)	C8—C4—C3—C2	−177.58 (14)
C6—C5—C4—C3	−2.52 (19)	C3—C4—C8—C9	−177.58 (14)
C15—C14—C13—C12	−1.66 (19)	C3—C4—C8—C11	62.44 (18)
C16—C17—C12—O3	179.13 (11)	C3—C4—C8—C10	−56.87 (17)
C16—C17—C12—C13	−0.12 (17)	O6—Cu2—O3—C12	−138.98 (16)

Symmetry code: (i) −*x*+2, −*y*+1, −*z*.

### Hydrogen-bond geometry (Å, º)

**Table:**

*D*—H···*A*	*D*—H	H···*A*	*D*···*A*	*D*—H···*A*
C14—H14···O4^ii^	0.95	2.57	3.3225 (16)	136
C23—H23*B*···O2	0.98	2.43	3.0607 (18)	122

Symmetry code: (ii) *x*−1/2, −*y*+1/2, *z*−1/2.
